# Supplementary material for: The Evolution and Functional Roles of miR408 and Its Targets in Plants
Source: Int J Mol Sci. 2022 Jan 4;23(1):530. doi: 10.3390/ijms23010530 (PMC8745667; doi:10.3390/ijms23010530)
Supplement: Supplementary file 1 [file ijms-23-00530-s001.zip › Table S5.pdf]

Table S5 Experimental validated of predicted miR408 targets in plants

Species	Sequences		References
Arabidopsis thaliana	2/12 ↓<br>5'-CCAAGGGAAGAGGCAGUGCAU-3'<br>0|0||||||||||||||||00<br>3'-CGGUCCC UUCUCC GUCACGGU-5'	plantacyanin<br><br>miR408	[36]
	9/12 ↓<br>5'-ACCAGUGAAGAGGCUGUGCAG-3'<br>0||||0||||||||0|||||0<br>3'-CGGUCCC UUCUCCGUCACGUA-5'	LAC3<br><br>miR408	[36]
	4/4 ↓<br>5'-GCCGGUGAAGAGGCUGUGCAA-3'<br>|||0|0||||||||0|||||0<br>3'-CGGUCCCUUC UCCGUCACGUA-5'	LAC12<br><br>miR408	[36]
	1/12 ↓ 6/12 ↓<br>5'-CGCCGGUGAAGAGGCUGUGCAG-3'<br>|||0|0||||||||0|||||0<br>3'- CGGUCCCUUCU CCGUCACGUA-5'	LAC13<br><br>miR408	[36]
	1/8 ↓ 5/8 ↓<br>5'-GCCGGUGAAGAGGCUGUGCAA-3'<br>|||0|0||||||||0|||||0<br>3'-CGGUCCCUUC UCCGUCACGUA-5'	LAC12<br><br>miR408	[37]
	1/11 ↓ 6/11 ↓<br>5'-GCC GGUGAAGAGGCUGUGCAG-3'<br>|||0|0||||||||0|||||0<br>3'-CGGUCC CUUCUCCGUCAC GUA-5'	LAC13<br><br>miR408	[21, 37]
	8/10 ↓ 2/10 ↓<br>5'-CCAAGGGAAGAGGCAGUGCAU-3'<br>0|0|||||||||||||||||| 3'-CGGUCCCUUCUC CGUCACGUA-5'	plantacyanin<br><br>miR408	[21]
	2/20 6/20 ↓↓<br>5'-CCAAGGGAAGAGGCAGUGCAU-3'<br>0|0|||||||||||||||||| 3'-CGGUCCCUUCUCCGUCACGUA-5'	plantacyanin<br><br>miR408	[16]
	1/4 ↓<br>5'-CCAAGGGAAGAGGCAGUGCAU-3'<br>0|0|||||||||||||||||| 3'-CGGUCCCUUCUCCGUCACGUA-5'	plantacyanin<br><br>miR408	[20]
	1/4 ↓<br>5'- GCCAGGGUGGAGUCAGUGCUU -3'<br>|||||||00|||0||||||0| 3'-CGGUCCCUUCUCCGUCACGUA-5'	UCC2<br><br>miR408	[20]

	1/4 3/4<br>5'-ACCAGUGAAGAGGCUGUGCAG-3' LAC3<br>o||||o||||||||o|||||o<br>3'-CGGUCCC UUCUCCGUCACGUA-5' miR408<br><br>4/4<br>3'-GCCGGUGAAGAGGCUGUGCAA-5' LAC12<br>|||o|o||||||||o|||||o<br>5'-CGGUCCCUUC UCCGUCACGUA-3' miR408	
	3/4<br>3'-GCC GGUGAAGAGGCUGUGCAG-5' LAC13<br>|||o|o||||||||o|||||o<br>5'-CGGUCC CUUCUCCGUCAC GUA-3' miR408	
	1/10 2/10 6/10<br>5'- CCCAGGGAAGAGGCAGUGCAG-3' plantacyanin<br>o|||||||||||||||||||| 3'-CGGUCCCUUCUC CGUCACGUC-5' miR408	[38]
	12/13<br>5'- C UCGGGGAAGAGGCAGUGCAA-3' UCL8<br>oo |o|||||||||||||||| 3'-CGGUCCCUUCUCCGUCACGUC-5' miR408	[26]
	12/13<br>5'- GCCAGGAUAGAGGCAGUGCAU-3' PLC-like1<br>||||||oo||| |||||||||o<br>3'-CGGUCCCUUCU CCGUCACGUC-5' miR408	[20]
Oryza sativa	6/6<br>5'-CCACCTGCCAGGATAGAGGC AGTGCATATGATT-3' UCL30<br>||||||oo||oo||||||| 3'-CGGTCCCT TCT CCG TCACGTC-5' miR408	[39]
	Cleavage site<br>5'-CGGUCCCUUCUCCGUCACGUC-3' DSHCT<br>||||o|||o|||||o|||| 3'-GCCAAGGACGAGGCGGUGCA-5' miR408	[40]
	1/6<br>5'-GCCGGUGAAGAGGCUGUGCAA-3' LAC3-like1<br>|||o|o||||||||o|||||o<br>3'-CGGUCCCUUCUCCGUCACGUC-5' miR408	[20]
Nicotiana tabacum	2/6 1/6<br>5'-GCUAGGCGAGAGGCAGUGCUU-3' UCC-like1<br>||o|||oo|||||||||||o| 3'-CGGUCCCU UCUCCGUCACGUA-5' miR408<br><br>3/6<br>5'-GUCAGGGAAGAAGCAGUGCAA-3' PLC-like1	[20]

	3'-CGG UCCCUUC UCCGUCACGUA-5' miR408<br>5/6<br>5'-ACCAGUGAAGAGGCUGUGCAU-3' LAC12-like1<br>3'-CGGUCCCU UCUCCGUCACGUA-5' miR408	
Triticum aestivum	6/28<br>5'-GCCGGGGTAGGCGGAGGGCAG-3' TaTOC-A1<br>3'-CGGUCCCUUCUCCGUCACGUC-5' miR408<br>8/28<br>5'- G CCGGGGTGGGGGGAGGGCAG-3' TaTOC-B1<br>3'-CGGUCCCUUCUCCGUCACGUC-5' miR408<br>14/28<br>5'-GCAGGGGCAGGGGCAGGGCAG-3' TaTOC-D1<br>3'-CGGUCCCUU CUCCGUCACGUC-5' miR408	[24]
Salvia miltiorrhiza	9/13 1/13<br>5'- AAGCAACCCCAGTGAAGAGGCTGTGCAAGACCC-3' LAC3<br>3'-TCGGTCCCTTCTCCGTCACGT-5' miR408<br>13/15<br>3'-CAATCAACACCAGTGAAGAGGCTGTGCAAAACTC LAC18<br>3'-TCGGTCC CT TC TCCGTCACGT-5' miR408	[41]
Ipomoea batatas	11/21 8/21<br>5'-GCCGCCAGGGAAGAGGCAGAGCAGG-3' IbKCS<br>3'-UCGGUCCCUUCUCCGUC ACGU-5' miR408<br>7/7<br>5'-GUCGUGAGGGAAGAGGCAGUGCAGU-3' IbPCL<br>3'-UCGGUCCCUUCUCCGUCACGU5' miR408<br>17/20<br>5'-UCCGCCGGGGAAGAGGCGGUGGAAU-3' IbGAUT<br>3'-U CGGUCC CUUCUC CGUCACGU-5' miR408	[44]
Saccharum officinarum	8(10)<br>5'-GCCGGUGAAGAGGCUGUGCA-3' Diphenol oxidase laccase<br>3'-CGGUCCCUUCU CCGUCACGU-5' miR408	[64]
Medicago	8/17 9/17	[42]

truncatula	3’-GUAGAGAAACAUGGCUCUGGGAAGAGGCAGUGCAUUGGUU-5’ plantacyanin<br>3’-CGGUCCCU UCUCCGUCACGUA-5’ miR408	
Dimocarpus longan	2/7 5/7<br>ACCCAAAGTTCACAGCCATGAATTTGTTATTCAAGCTACACCAGGTGAAGAGGCTGTGCAGAA LAC12<br>CGGUCC C UUCUCCGUCACGUA miR408-3p	[65]

Vertical arrows indicate the 5’ termini of miRNA-guided cleavage products, as identified by 5’-RACE, with the frequency of clones shown. Perfect base pairing is shown as vertical dashes, whereas G:U wobble pairing is indicated by circles. PLC, plantacyanin, UCC, Uclacyanin, LAC, laccase, DSHCT, DOB1/SK12/helY-like DEAD-box Helicase, LOC_Os11 g07500, IbKCS, 3-ketoacyl-CoA synthase 4; IbPCL, Plantacyanins; IbGAUT, galacturonosyltransferase 7-like, TaTOC1, Triticum aestivum TIMING OF CABEXPRESSION 1.
